# Core values and principles of general practice and family medicine: perspectives of German GP residents—a cross-sectional study

**DOI:** 10.3389/fmed.2025.1495789

**Published:** 2025-02-26

**Authors:** Andreas Christian Dreher, Jonathan Ko, Christine Becker, Martina Bischoff, Christian Förster, Tanja Jähnig, Sandra Stengel, Attila Altiner, Simon Schwill

**Affiliations:** ^1^University Hospital Heidelberg, Department of Primary Care and Health Services Research, Heidelberg University, Heidelberg, Germany; ^2^University Hospital of Freiburg, Institute of General Practice, Medical Center, University of Freiburg, Freiburg, Germany; ^3^Institute of General Practice and Interprofessional Care, University Hospital Tübingen, Tübingen, Germany; ^4^Institute of General Practice, University Hospital Ulm, Ulm, Germany

**Keywords:** core values, core principles, family medicine, FM training, Germany, reflective practitioner

## Abstract

**Background:**

The core values and principles of general practice (GP) and family medicine (FM) have been described by various international scientific societies, including the World Organization of National Colleges, Academies, and Academic Associations of General Practitioners/Family Physicians (WONCA). These values and principles, such as continuity of care, a bio-psycho-social approach, and hermeneutic case understanding, are also integrated into FM training programs. The aim of this study was to investigate the knowledge and perspectives of FM trainees regarding the core values and principles of FM.

**Methods:**

In a cross-sectional study, new participants of the postgraduate FM training program KWBW Verbundweiterbildung plus© were asked to complete a self-developed questionnaire on their educational experiences, attitudes toward, and knowledge of core values in GP/FM. Specifically, participants were asked to identify the core values and principles associated with GP/FM. Qualitative analysis was used to explore the answers. Additionally, participants were required to define a set of core values, which were then analyzed semi-quantitatively and rated as correct, semi-correct, wrong, or unknown.

**Results:**

Out of a total of *n* = 303 trainees, *n* = 250 completed the questionnaire. The majority (*n* = 194) were in their third year of training. A third of the participants reported having studied core values in the past. The participants identified several core values and principles associated with GP/FM. The practical relevance and confirmation of becoming an FM doctor were well-evaluated.

**Conclusion:**

The study identified deficits in the active and passive knowledge of GP/FM core values among GP residents. An educational compact intervention about GP/GM core principles and values proved successful in its realization and implementation. To become a reflective practitioner in FM, GP residents must engage in self-reflection on evidence-based medicine, attitudes, core values, and principles. Therefore, core values should be addressed at the beginning of FM training and constantly referred to within the longitudinal curriculum. Family physicians should be continuously empowered to explicitly reflect on and discuss the core principles that shape their professional identity.

## Introduction

The core values of general practice/family medicine (FM) provide a framework for the professional role and responsibilities of general practice (1). They have been described by various international scientific societies, including the World Organization of National Colleges, Academies, and Academic Associations of General Practitioners/Family Physicians (WONCA). Within different countries, FM core values are ranked differently but pertain to the same nexus (2–4). The professional definition of the German College of General Practitioners and Family Physicians (DEGAM) outlines a set of FM core values, including a bio-psycho-social approach, unselected patients, preventable dangerous or irreversibly harmful course of disease, wait-and-see attitude, experienced anamnesis, and hermeneutic case understanding. In 2022, the Göktaş definition of family medicine/general practice emphasized the value of hermeneutic case understanding by adding the broad authority and perspective it offers to the family physician, complementing the core competencies and principles outlined in the previous WONCA definition (6). Furthermore, the CanMED role model plays an important role in FM and medical education (5).

There is limited evidence about the knowledge of FM core values and principles among FM trainees. The FM core principles are typically taught through reflection and practice (7), as well as in seminars built around overarching topics (8). Teaching principles and values in general are outlined for basic medical education (9, 10), with a suggestion for a practical and theoretical model (11).

Defining and vocalizing core values is an important foundation for using these values in daily evidence-based FM routines (12) and can help maintain course in a constantly changing world (13). Knowledge of the core principles of FM is listed as a learning objective in the postgraduate training curriculum of the German Federal Medical Association (14). The deep learning and decisive application of FM core principles in daily practice can make healthcare systems more efficient and effective (12).

The German education guidelines for family physicians (14) require 5 years of training with mandatory on-ward training in internal medicine (12 months) and training at an FM surgery (24 months), as well as 24 months of further training in other elective rotations and in patient-related specialist rotations (15). Graduates from other specialties, such as surgery, neurology, or internal medicine, may enter FM training through lateral entry with a 2-year curriculum (16). The KWBW Verbundweiterbildung PLUS© is the first German voluntary competency-based training program to offer a seminar curriculum including educational compact interventions (17–20) as well as structured mentoring and the possibility of regional clinical rotations across Baden-Württemberg for FM trainees (21). At the beginning of this program, attendees have to participate in a 2-day starter course with two seminars (2 × 90 min) about FM core values and principles.

Within this cross-sectional study, mixed methods were used to achieve the aim of this study. These were

- to explore the current understanding of FM core values by trainees
- to evaluate an educational compact intervention on FM core values and principles

## Methods

### Type of study, setting, and participants

A cross-sectional analysis surveying FM trainees in Baden-Württemberg (Germany) was conducted between September 2020 and December 2021. All FM trainees entering the FM training program *KWBW Verbundweiterbildung plus* have to participate in a two-day starter course. Starter courses have a maximum of 25 attendees, are offered 8–10 times per year, and are performed either in person or online. In the survey period, all 303 registered attendees were invited to participate in the study, excluding those residents who had been part of the study team.

### Survey

In a literature search, a validated tool to question the core values and principles of FM trainees could not be found. A first proposal for the questionnaire was developed by the project team familiar with questionnaire development, including additional FM advice and target-group-specific input. It was first validated using the think-aloud technique (22). In this method, participants verbalize their thought processes while completing the questionnaire, providing insights into their cognitive strategies and decision-making. Second, the questionnaire was pre-tested with one cohort, leading to minor changes before it was finalized (Supplementary material 1). Questions included sociodemographic data and previous education in FM core values. Second, participants were asked to name core values and principles they consider important for FM (free-text). Third, participants were asked to actively define the core values and principles of FM according to the DEGAM (German Society for General and FM), including the bio-psycho-social approach, unselected patient population, preventable dangerous or irreversibly harmful course of disease, wait-and-see attitude, experienced medical history, hermeneutic case understanding, and CanMED role model. It was also possible to mark the terms as “unknown.” The explanations were categorized as correct, semi-correct (partially correct but with at least one inaccurate term), or wrong by the authors (AD and SiS).

### Data collection and data analysis

The questionnaire was paper-based and sent out to registered attendees 1 week before the start of the 2-day starter course. Attendees of online starter courses were provided with a stamped return envelope. At the beginning of the course, trainees were informed about the survey and invited to participate. They were given time to fill out the survey, with data collection taking place between November 2020 and December 2021. Participation in the KWBW program is generally voluntary; however, participating in the 2-day starter course at the beginning is mandatory for those wishing to enter the program. This results in potential volunteer bias, which is reduced by the mandatory participation and the high number of participants.

A descriptive analysis of quantitative items was performed. Free text sections (description of core values and principles) were analyzed qualitatively through an inductive process by two independent authors of the study team (AD and SiS), following the approach outlined by Kuckartz (23). The definition of core values and principles was analyzed semi-quantitatively and categorized as “correct, semi-correct, incorrect, and unknown” by the same researchers (AD and SiS). In cases of individual discrepancies, a third author was involved in the resolution process (JK).

### Intervention

Participants of this study took part in a seminar (two 90-min sessions) about core principles and core values in FM at the 2-day starter course, which is compulsory at the beginning of the FM training program *KWBW Verbundweiterbildung plus* (Supplementary material 2). The 2-day starter course itself includes eight 90-min sessions. In seminar units on medical topics, reference is made to the core principles and values, and the participants reflect on their actions in this regard, such as headaches and chest pain. The course was evaluated using an online evaluation questionnaire (SurveyMonkey, SurveyMonkey Europe UC, Dublin, Ireland) with a Likert scale (1–6), covering the following categories: practical relevance, satisfaction with content, didactic method, and possibility of participation.

## Results

### Sociodemographic data

Of *n* = 303 invited residents, *n* = 250 (response rate: 82.5%) completed the survey. Table 1 depicts participants’ sociodemographic data. The majority (*n* = 194) were in their third year of training (average 3.13 years), and the majority of lateral entry trainees (*n* = 56) were in their first of 2-year training (average: 1.38). A total of 80% were currently placed in an FM practice.

**Table 1:** Sociodemographic data of family medicine trainees participating in an educational compact intervention on FM core principles and values (*n* = 250).

*n* = 250		
Sex	Male	56 (22.4%)
	Female	194 (77.6%)
Age (years)	Median (IQ)	35 (31;37)
Year of training	Average	3.21 (SD = 1.57)
Current rotation placement	Family medicine	200 (80%)
	Hospital	50 (20%)
Training	Direct entry	194 (77.5%)
	Lateral entry	56 (22.5%)

### Previous education in family medicine core values

A total of 82 participants (33.2%) stated they studied FM core values in depth previously. A total of 73 participants (29.2%) reported receiving education in FM core values during medical school, while 9 participants (3.6%) stated that they had participated in a seminar about FM core values after graduation from medical school. Out of 100 statements (with multiple answers possible), the most frequently mentioned learning resources were medical school, FM books, final year placement in FM, and practical activities during FM training placement.

### Core values and principles in FM

In a free-text section (with 10 answers possible), participants were asked to describe what they associate with FM’s attitude and core values. A wide variety of responses were given, reflecting the scope of work in FM. The most commonly mentioned concepts refer to prevention, coordination, a comprehensive approach, and long-term patient care. On average, the answers referenced three core values from the WONCA definition and two FM core values from the DEGAM definition of FM. In Table 2, the answers are categorized semi-quantitatively by two researchers (AD and SiS). In cases of individual discrepancies, a third author was involved in the resolution process (JK).

**Table 2:** Open text answers questioning core values and core principles of family medicine trainees participating in an educational compact intervention on FM Core Principles and Values.

*n* = 1,198	DEGAM	%	WONCA	%
Statements with reference to core values and principles	(a) Biopsychosocial approach	106 (8.9%)	Person-centered care	261 (21.7%)
	(b) Unselected patients	63 (5.3%)	Continuity of care	149 (12.4%)
	(c) Preventably dangerous or irreversibly harmful course of disease (red flags)	60 (5.0%)	Science-oriented care	38 (3.2%)
	(d) Wait-and-see attitude	52 (4.3%)	Cooperation in care	86 (7.2%)
	(e) Experienced anamnesis	28 (1.8%)	Equity of care	6 (0.5%)
	(e) Hermeneutic case understanding	4 (0.23%)	Community-oriented care	205 (17.1%)
			Professionalism in care	4 (0.23%)

Clustered answers compared to DEGAM and WONCA definition. DEGAM, German College of General Practitioners and Family Physicians; WONCA, World Organization of National Colleges, Academies, and Academic Associations of General Practitioners/Family Physicians.

Following are two representative quotes from this section showing the perceived diversity and complexity of family medicine:

*“diagnostic uncertainty, detecting red flags, broad decision-making competence, long-term patient care, importance of doctor-patient relationship, contact person in many life situations, coordination function, prevention, vaccination counseling, individualization of guidelines” (#62).*

Another participant stated further aspects of FM practice:

*“First contact, easy access to health care, long-term relationship/long-term support, comprehensive bio-psycho-social care, coordination of care, availability of a specific decision-making process determined by the prevalence and incidence in the population, promotion of health and well-being through appropriate and effective interventions, early stage diseases with undifferentiated symptoms and potentially urgent interventions” (#91).*

### Current knowledge of FM’s core values

Widely correct explanations were given for the bio-psycho-social approach (*n* = 186 out of 250), unselected patients (*n* = 191 out of 250), and avoidable life-threatening conditions (*n* = 221 out of 250). Mixed performance was observed with the correct explanation of experiencing anamnesis (*n* = 174 out of 250). Hermeneutic case understanding and CanMED role model were mainly categorized as “unknown” (both *n* = 215 out of 250) by the majority of participants (Table 3). Representative explanations are shown in Table 4.

**Table 3:** Categorization of answers defining terms of core principles of FM by FM trainees participating in an educational compact intervention on FM core principles and values about FM practice.

*n* = 250	Wrong	Correct	Semi-correct	Unknown	No statement
Biopsychosocial approach	0 (0%)	186 (74.4%)	3 (1.2%)	43 (17.2%)	18 (7.2%)
Unselected patients	0 (0%)	191 (76.4%)	4 (1.6%)	40 (16%)	15 (6%)
Preventably dangerous or irreversibly harmful course of diseases	1 (0.4%)	221 (88.4%)	2 (0.8%)	14 (5.6%)	12 (4.8%)
Wait-and-see attitude	0 (0%)	218 (87.2%)	4 (1.6%)	15 (6%)	13 (5.2%)
Experienced anamnesis	0 (0%)	174 (69.6%)	12 (4.8%)	48 (19.2%)	16 (6.4%)
Hermeneutic case understanding	0 (0%)	17 (6.8%)	0 (0%)	215 (86%)	18 (7.2%)
CanMED role model	0 (0%)	18 (7.2%)	0 (0%)	215 (85.6%)	18 (7.2%)

**Table 4:** Selected explanations of family medicine core principles by FM trainees participating in an educational compact intervention on FM core principles and values about FM practice.

Category	# (Participant)	Selected explanations
(a) biopsychosocial approach	1 (#62)	*“Health as a result of a balance of a healthy body, soul, and psyche, as well as social environment”*
	2 (#177)	*“Consideration of mental, social and somatic factors of the patient”*
(b) Unselected patients	1 (#32)	*“First contact with a broad and unknown spectrum of symptoms”*
	2 (#141)	*“from all social layers with all diseases”*
	3 (#201)	*“unselected from all age groups and social classes”*
(c) Avoidable life-threatening conditions	1 (#66)	*“Due to severe symptoms immediate action is needed”*
	2 (#123)	*“Symptoms showing possible acute life-threatening situation”*
(d) Wait-and-see attitude	1 (#44)	*“no secure assignment to one disease, wait and see attentively”*
	2 (#173)	*“no intervention at first, follow up, whether an intervention is needed at a later point in time”*
	3 (#204)	*“Unclear symptoms without red flags, observe and wait together with the patient”*
(e) Experienced anamnesis	1 (#89)	*“Consideration of the past of the patient as well as previous experiences with the patient”*
	2 (#117)	*“Knowing the patient and living conditions, long-term support over years”*
	3 (#204)	*“Information beyond medical anamnesis (e.g., mental, job)”*
(f) Hermeneutic case understanding	1 (#64)	*“Consideration of all aspects of the patient in the interpretation of symptoms and findings”*
	2 (#165)	*“Interpretation with consideration of different aspects of the patient”*
	3 (#239)	*“Understanding patient case in its complexity taking all influencing factors into account”*
(g) CanMED role model	1 (#134)	*“Profession of the physician is divided into 6 skills. A physician should possess these skills to fulfill the physician’s role”*
	2 (#211)	*“Describes the role of the FM physician apart from medical knowledge”*

### Basic evaluation of training in core values (intervention)

Participants were asked to evaluate the 2-day starter course with separate evaluation sections for the different seminars. There was a seminar (two 90-min sessions) about FM core principles and values, explaining and reflecting on them based on daily FM practice and clinical examples. The seminar received good evaluation in all categories (Table 5). The categories and their corresponding ratings were: practical relevance (1.71), satisfaction with content (1.93), educational methods (1.94), the possibility of participation (1.86), and confirmation to become an FM doctor (1.74).

**Table 5:** Evaluation of an educational compact intervention on core principles and core values by FM trainees.

*n* = 232	Likert scale (1 = very good; 6 = very bad)
Practical relevance	1.71 (SD = 0.69)
Satisfaction with content	1.93 (SD = 0.83)
Educational methods	1.94 (SD = 0.87)
Possibility for participation	1.86 (SD = 0.72)
Confirmation to become an FM doctor	1.74 (SD = 0.71)

## Discussion

To the best of the authors’ knowledge, this is the first study to analyze the knowledge of FM core values among trainees in Germany. The study showed that education in FM principles and values at medical school and during postgraduate training cannot be taken for granted and needs to be implemented in the curricula. An educational course on principles and core values was widely accepted and rated well. All the objectives of the study were achieved.

There was a strong implicit understanding of FM core principles and values, while, on the other hand, there was a lack of explicit knowledge of specific terms that define these core principles and values. In the authors’ view, explicit knowledge and vocation help to internalize and reflect principles and values and promote advocacy for FM. This phenomenon is also described in nursery education (24). Full explicit knowledge of FM core values can serve as a valuable guidance for general practitioners. Explicitly naming FM core principles as a learning objective, such as in the postgraduate training curriculum of the German Federal Medical Association (14), seems both progressive and desirable to us. In sum, we lack implementation of teaching FM core principles and values in medical education.

The data show that not all participants experienced comprehensive education in FM core principles and values during medical school. The majority of participants are in the advanced status of their training, and various FM trainees report no previous specific training in FM core principles and values. We can therefore conclude that explicit training in the core values of FM cannot be taken for granted, and it would be wrong to presume knowledge of FM core values as known (8). Rarely specific teaching of FM core values and principles is described, which underlines the importance of the seminar. Educational interventions focused on FM core values can increase explicit and specific knowledge, skills, and attitudes in FM. Cost-effective and time-efficient educational compact interventions on a variety of topics have proven successful in fostering the competencies of FM trainees (17–20). The educational compact intervention about core values was well-evaluated especially in the categories of practical relevance and confirmation to become an FM doctor. Considering the four stages of competence (25), it is important to learn the core principles and values at the level of conscious incompetence as well as at the level of conscious competence. This approach ensures the development of full knowledge at the level of unconscious competence level for future FM doctors.

The explicit knowledge of FM core values enables more specific personal reflection, which can be a relevant factor in becoming a reflective family physician (26). Having a specific framework of FM principles can be a helpful reference at the beginning of the FM rotation where a transformation from a clinical attitude toward a primary care attitude is needed (27). Furthermore, it could foster advocacy for FM (1). The World Federation for Medical Education (WFME) training curriculum for FM trainers needs to integrate core principles and concepts of FM (28). There are also indications for new values, for example for employed FM doctors (29). At KWBW Verbundweiterbildung plus, we have specific training in FM core principles and values, and references are continually made during the whole 5-year program.

There is an ongoing constructive and valuable discussion about identifying and ranking the core principles and values of FM (30). In this study, we used the German definition and assumed that, with a different definition in another country, a similar gap between implicit and explicit knowledge would likely be shown. In view of this, together with the good rating in terms of practical relevance, we see a need for further implementation of the teaching of core values at both pre- and post-graduate levels.

We think that longitudinal medical curricula based on core principles and values, with educational compact interventions at the beginning and corresponding further learning objectives, can also be effective in other disciplines and other settings with correlating specific values. We suggest that such longitudinal curricula can also be integrated into existing medical curricula in other countries. Further didactic implementation can be an integration of a spiral curriculum, practice- and case-based learning, and longitudinal reflection (31, 32).

### Strengths and limitations

There are several limitations to this study. The collected data are from trainees in Baden-Württemberg, a federal state of Germany with approximately 11.2 million inhabitants. A total of 250 participants (>80% of the annual cohort) completed the questionnaire. However, this may be representative of the whole of Germany but not of Europe or FM trainees in general. The participants were trainees at an advanced stage of their training, representing a progressed training status, which permits reliable conclusions about experienced education within training and medical school.

At the time of data collection, the majority of residents had started their outpatient rotation in FM practice. However, the data do not show knowledge after 2 years of training in FM. Participation in the KWBW is voluntary, therefore participants may be highly motivated FM trainees with a strong emphasis on good training. Participation in the course about core values and principles, however, is compulsory to enter the program.

## Conclusion

In conclusion, to support professional identity formation in FM, it is necessary to ensure explicit learning, discussion, and reflection on FM core values during training. A compact educational intervention in FM core values and principles was well evaluated by FM trainees, especially in the category of practical relevance. The results of this study align with the research objectives and provide comprehensive insights into training FM core values and core principles.

Awareness of the core principles and values of FM can empower trainees, encourage advocacy for FM, and enhance its reputation. Training programs in FM should implement the core principles of FM into seminars at the very beginning of the training curriculum and should recur to them later. Educators should integrate reflection on core values and principles alongside the teaching of knowledge and skills. This allows for continuous self-reflection during the postgraduate training as well as an ongoing, lifelong process. Policymakers should support the development and implementation of longitudinal curricula that emphasize core values and principles, evidence-based medicine, and professional identity formation.

Future studies should investigate the implementation of FM core values in curricula at medical school, during training, and in continuous medical education. Further interventions should prioritize the implementation of longitudinal curricula that focus on professionalism and attitudes. Studies should focus on a pre–post design with follow-up on knowledge and professional identity formation, attitudes toward core principles and values, and potential effects on self-reflective practice. A longitudinal evaluation with a built-in progression model of attitudes toward core values and principles and professional identity formation could assess long-term effects. In addition, its possible long-term effects on daily primary care practice need to be examined. Longitudinal and qualitative studies are needed to further explore how successful interventions support FM trainees to gain FM core values and principles as unconscious competence in the daily practice of FM.

## Data Availability

The datasets presented in this article are not readily available because as required by the European Data Protection law the participants were informed of the privacy policy and agreed that the pseudonymized data would be available to the project team and would be stored at the study center. Request to access the datasets should be directed to Simon Schwill (simon.schwill@med.uni-heidelberg.de).
