# Supplementary material for: Core values and principles of general practice and family medicine: perspectives of German GP residents—a cross-sectional study
Source: Front Med (Lausanne). 2025 Feb 26;12:1495789. doi: 10.3389/fmed.2025.1495789 (PMC11898739; doi:10.3389/fmed.2025.1495789)
Supplement: Supplementary file 1 [file Table_1.docx]

Supplementary Material

# Supplementary Questionnaire

Have you attended a course on “Core Principles of General Practice” after completing medical school? 🡪 yes / no

If yes: Who offered this course? (multiple answers possible) 🡪 university, medical faculty / university, other faculty / congress General Practice/Family Medicine / *KWBW Verbundweiterbildung^plus^* / Medical Association / others (free text answers)

Did you ever take part in a course on “Core Principles of General Practice” during your medical studies? 🡪 yes / no

If yes, at which university did you study? 🡪 free text answer (city, country)

Have you ever taken a closer look at the core principles of General Practice/Family Medicine?

If yes, how? (free text answer in keywords)

When did you complete your medical studies? 🡪 year

Which specialist training did you start after your studies (aspired specialist)? 🡪 General Practice/Family Medicine / other (free text answer)

In which professional year (since completing your studies) did you decide to train as a specialist in General Practice/Family Medicine? 🡪 year (if you decided during medical school or earlier, please answer with “0”)

Why did you decide to train as a general practitioner? 🡪 Please name 3 main reasons in key words

Was there a personal initiating event? 🡪 initiating event: free text answer

Are you doing your specialist training in general medicine as a lateral entry? 🡪 yes / no

If yes, which specialist training have you completed? 🡪 free text (f.ex. anesthesiology)

What do you consider to be the Core Values und Principles of General Practice/Family Medicine? 🡪 Please name up to 10 key words (free text answer)

If possible, please explain the following term (if you have not heard the term before, please not “unknown”): biopsychosocial approach (free text answer)

If possible, please explain the following term (if you have not heard the term before, please not “unknown”): unselected patients (free text answer)

If possible, please explain the following term (if you have not heard the term before, please not “unknown”): irreversibly harmful course of diseases (free text answer)

If possible, please explain the following term (if you have not heard the term before, please not “unknown”): wait-and-see attitude (free text answer)

If possible, please explain the following term (if you have not heard the term before, please not “unknown”): experienced anamnesis (free text answer)

If possible, please explain the following term (if you have not heard the term before, please not “unknown”): hermeneutic case understanding (free text answer)

If possible, please explain the following term (if you have not heard the term before, please not “unknown”): CanMEDs role model (free text answer)

Your gender? 🡪 female / male / diverse

When were you born? 🡪 year

What year of training are you in? 🡪 1-5 years full-time equivalent?

When did you enter the *(KWBW) Verbundweiterbildung^plus^* 🡪 month/year

What stage of training are you in? 🡪 inpatient sector / outpatient sector

Do you have any comments or suggestions for the questionnaire? 🡪 (free text)

Thank you for your participation!

**
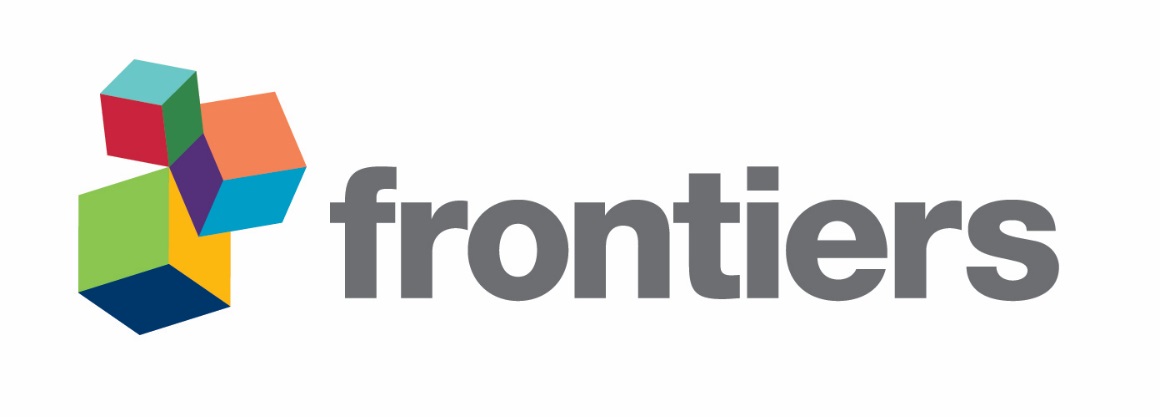
**
