# Supplementary material for: Core values and principles of general practice and family medicine: perspectives of German GP residents—a cross-sectional study
Source: Front Med (Lausanne). 2025 Feb 26;12:1495789. doi: 10.3389/fmed.2025.1495789 (PMC11898739; doi:10.3389/fmed.2025.1495789)
Supplement: Supplementary file 2 [file Table_2.docx]

**Seminar module (#33/62): Core values and Core principles / uncertainty**

Learning aims: (“at the end of the seminar I am able…”):

- To explain the definition of General Practice / Family Medicine according to DEGAM
- To relate my decisions in Family Medicine with the principles of FM: preventably dangerous or irreversibly harmful course of disease (red flags), experienced anamnesis, wait- and – see attitude, unselected patients with low prevalence, filtering function, step-by-step diagnostics
- To choose the adequate decision aids (guideline, scores etc.)
- To critically reflect the significance of intuition and subjectivity
- To justify diagnostic uncertainty in reflection of the Bayes-Theorem (pre- and post-test-probability)
- To reflect on own handling of uncertainty

Inverted classroom: (residents get this information 2 weeks in advance via mail)

- Self-assessment of the learning aims in the competence-based curriculum
- Please reflect before the seminar:
  - Reflect your uncertainty in decision making as well as after therapeutic decisions
    - What is difficult to endure?
    - What is easy to endure?

Schedule: 90 minutes, presence or digital, approx.. 25 participants

| **What time?** | **What?** | **How?** | **What for?** | **Who?** |
| --- | --- | --- | --- | --- |
| 15 min plenum | Introduction and link to preparation (inverted classroom) | Introduction of person and topic  2 polls (shouting out or online poll tool)  definition of FM/GP, over- and underuse, health disparities, dealing with uncertainty, preparation | Arriving  Secure same information base in the group  Explanation of terms  Triggering attention for new things |  |
| 5 min marble group | As two or three | Reflection about dealing with uncertainty | Personal reflection (dealing with uncertainty) |  |
| 15 min small groups | Group work | 4 groups   1. Doctor-patient relationship, continuity of care, bio-psycho-social approach 2. Experienced anamnesis, low prevalence 3. preventably dangerous or irreversibly harmful course of disease (red flags), wait-and-see attitude 4. filter and coordination function / gatekeeping function | Developing content |  |
| 10 min plenum | Theme 1: Doctor-patient relationship, continuity of care, bio-psycho-social approach | Presentation of small group, discussion, exchange, supplementary input slides | ensure that everyone knows the FM core principle and reflects on own medical practice |  |
| 10 min plenum | Theme 2: Experienced anamnesis, low prevalence | Presentation of small group, discussion, exchange, supplementary input slides | ensure that everyone knows the FM core principle and reflects on own medical practice |  |
| 5 min short break |  | activity |  |  |
| 10 min plenum | Theme 3: preventably dangerous or irreversibly harmful course of disease (red flags), wait-and-see attitude | Presentation of small group, discussion, exchange, supplementary input slides | ensure that everyone knows the FM core principle and reflects on own medical practice |  |
| 10 min plenum | Theme 4: filter and coordination function / gatekeeping function | Presentation of small group, discussion, exchange, supplementary input slides | ensure that everyone knows the FM core principle and reflects on own medical practice |  |
| 5 min plenum, short | Closing seminar session with link to the next session | How to apply FM principles and values in general practice | Next session: How to links Core Principles and Core Values with Evidence-based Medicine |  |

**Seminar module (#34/62): Core values and Core principles / evidence-based medicine / over- and underuse**

Learning aims: (“at the end of the seminar I am able…”)

- To understand maluse in medicine with concrete examples
- To reflect the DEGAM-guideline *Protection from over- and underuse – choosing together* with concrete examples if my own medical practice
- To name examples for underuse: healthy disparities and vulnerable patient groups
- To explain the principles of evidence-based medicine

Inverted classroom: (residents get this information 2 weeks in advance via mail)

- Self-assessment of the learning aims in the competence-based curriculum
- Please reflect before the seminar:
  - Reflect your information sources in daily medical practice regarding evidence and validity
    - What is helpful for you?
    - Where would you with for more security?

Schedule: 90 minutes, presence or digital, approx. 25 participants

| **What time?** | **What?** | **How?** | **What for?** | **Who?** |
| --- | --- | --- | --- | --- |
| 2 min plenum | Introduction and link to topic | How can I apply FM Core Values and Principles in my medical practice? | Arriving  Triggering attention for new things |  |
| 5 min plenum | Reflection in plenum | What are your thoughts, concerns, ideas, feelings? | reflection |  |
| 5 min marble group | As two or three | Reflection about dealing with uncertainty | Personal reflection (dealing with uncertainty) |  |
| 8 min plenum | Input / repetition | What is evidence-based medicine? |  |  |
| 15 min small groups | Group work | 4 groups   1. Where did I use EBM successfully? 2. Over- and undersupply, health disparities 3. PSA-Screening 4. Upper respiratory system infections/symptoms and antibiotic treatment 5. Feeling tired | Developing content,  Reflect own experiences |  |
| 10 min plenum | Theme 1: Where did I use EBM successfully? | Presentation of small group, discussion, exchange, supplementary input slides | ensure that everyone knows EBM and FM core principle and reflects on own medical practice; application of guidelines |  |
| 10 min plenum | Theme 2: Over- and undersupply, health disparities | Presentation of small group, discussion, exchange, supplementary input slides | ensure that everyone knows EBM and FM core principle and reflects on own medical practice; application of guidelines |  |
| 10 min plenum | Theme 3: PSA-Screening | Presentation of small group, discussion, exchange, supplementary input slides | ensure that everyone knows EBM and FM core principle and reflects on own medical practice; application of guidelines |  |
| 10 min plenum | Theme 4: Upper respiratory system infections/symptoms and antibiotic treatment | Presentation of small group, discussion, exchange, supplementary input slides | ensure that everyone knows EBM and FM core principle and reflects on own medical practice; application of guidelines |  |
|  | Theme 5: Feeling tired | Presentation of small group, discussion, exchange, supplementary input slides | ensure that everyone knows EBM and FM core principle and reflects on own medical practice; application of guidelines |  |
| 10 min plenum | Discussion / open questions / vision | Open questions?  Plenum discussion | Answering questions  Capturing potential resistance in the group |  |
| 5 min plenum, short | Closing seminar session with link to the next session | How to ensure this continually?  Motivation to become a reflective practitioner (continuous professional development) | How can I implement all this? |  |
